# Adaptive Party Choice of Low-Ranking Males in Fission–Fusion Dynamics of Chimpanzees in Kalinzu Forest Reserve, Uganda

**DOI:** 10.3390/ani12172240

**Published:** 2022-08-30

**Authors:** Shohei Shibata, Takeshi Furuichi, Chie Hashimoto

**Affiliations:** 1 Wildlife Research Center, Kyoto University, Inuyama 484-8506, Japan; 2 Japan Society for the Promotion of Science, Kojimachi, Chiyoda-ku, Tokyo 102-9983, Japan

**Keywords:** *Pan troglodytes*, aggression, fission-fusion dynamics, party size, dominance rank, Kalinzu Forest Reserve

## Abstract

**Simple Summary:**

Various mammalian species, including primates, exhibit fission–fusion dynamics (FFD), which alters the size of subgroups through splitting and merging. Among primates, chimpanzees (*Pan troglodytes*) have been particularly studied as a species that exhibits a high degree of FFD, wherein members of the same group form temporary subgroups (parties) that vary in both size and composition. Most studies on the FFD of chimpanzees have focused on its role in reducing food competition among individuals. In this study, we focused on the association patterns of each adult male chimpanzee and examined whether the social factors, including male dominance rank and aggression from other males, affect their party attendance behavior. Low-ranking males spent more time alone than other males when mating opportunities were absent. When females were available for mating, males of all ranks showed a similar party attendance behavior. The aggression increased with the number of males in the party, and low-ranking males received more aggression than higher-ranking males. These results suggest that low-ranking males frequently traveled alone to avoid aggression from other males unless they attended parties to seek mating opportunities. The FFD seemed to offer alternative tactics for low-ranking males to balance the costs and benefits of attending parties.

**Abstract:**

Several studies have examined factors that regulate fission–fusion dynamics (FFD) in chimpanzee communities, such as receptive females, predation risks, and food availability. However, the effects of these factors vary between populations. In this study, we conducted focal animal observations of adult males in the M group in Kalinzu to examine the influence of male dominance rank, aggression from other males, the presence of females exhibiting maximum sexual swelling (MS), and fruit abundance on male tendencies of party attendance. We found that low-ranking males spent more time alone than other males when females with MS were absent. In contrast, when females with MS were present, males of all ranks showed a similar tendency of party attendance. We also found that the aggressive interactions increased with the number of males irrespective of the presence or absence of females with MS, and low-ranking males attracted aggression more frequently than higher-ranking males. These results suggest that low-ranking males frequently ranged alone to avoid aggression from other males unless they attended parties to seek mating opportunities. We conclude that low-ranking males have alternative tactics to balance the costs and benefits incurred or gained when attending parties.

## 1. Introduction

Various mammalian species, including dolphins, elephants, giraffes, hyenas, and some primate species, exhibit fission–fusion dynamics (FFD), which alters the size of subgroups by splitting and merging [1,2,3,4,5,6]. Fission–fusion dynamics allow the adaptive adjustment of subgroup size to reduce feeding competition and improve foraging efficiency [7,8,9,10]. Among primates, chimpanzees (*Pan troglodytes*) have been particularly studied as a species that exhibits a high degree of FFD, wherein members of the same group form temporary subgroups (parties) that vary in both size and composition [11,12].

Research on the FFD of chimpanzees has revealed several factors that drive individuals to gather into relatively large parties, such as the risk of predation, proximity of neighboring groups, and presence of receptive females [13,14,15,16,17]. In contrast, research on factors that constrain maximum party sizes has mainly focused on ecological factors, such as fruit abundance and distribution [13,18,19,20]. However, the effects of these factors on FFD vary among populations. Chimpanzees have also shown flexibility in party size at study sites where fruit abundance varies relatively little and does not seem to constrain maximum party size [17,20,21].

In addition to the factors that form FFD, potential options in party participation based on the costs and benefits that the FFD might offer to the individuals should be investigated to improve the understanding of their social structure. Chimpanzees are also known to exhibit intense intragroup aggression [4,22,23], and social tensions might prevent individuals from gathering. In fact, several studies have suggested that female chimpanzees with young infants face potential costs from male infanticide and that this influences their decision about participating in parties [24,25,26]. Similarly, especially for low-ranking males, leaving a party during increased social tension among party members could be an adaptive strategy, as chimpanzees display linear dominance hierarchies, and low-ranking males are often subjected to aggression [23]. However, the effect of male aggression on the party attendance of low-ranking chimpanzees has received limited research attention. Investigating the potential role of the FFD is essential for understanding the mechanisms shaping their social systems. In this study, we hypothesized that the FFD may offer alternative tactics for low-ranking males to mitigate costs caused by disadvantages when competing for survival and reproduction against other adult males. We made the following three predictions from this hypothesis: (1) low-ranking males range alone or in small parties more frequently than higher-ranking males, (2) when there are no receptive females in the group, low-ranking males spend more time ranging alone than when there are receptive females in the group, and (3) low-ranking males attract more aggression when attending larger parties. To examine these predictions, we evaluated the effect of male dominance rank, party size, and the presence of receptive females on the party attendance of males and the frequency of received aggression within the parties. We also investigated the effect of fruit abundance to ensure that we accurately assessed the effect of social factors on the party attendance of chimpanzees.

## 2. Materials and Methods

### 2.1. Study Site and Subjects

In the study, we observed wild chimpanzees of the M group in the Kalinzu Central Forest Reserve, Uganda, where long-term research has been conducted since 1992 [27,28]. During this study, the group consisted of approximately 100 individuals, including 15 adult males aged over 15 years [29], 29 adult females, a few subadult females, and many juveniles and infants. All individuals of the M group were identified and habituated by the beginning of the study period. Our study subjects consisted of 10 adult males. The individual names, dominance ranks, estimated birth years, and age classes, defined in line with Goodall (1986), of each adult male are shown in Table 1. The dominance rank of males was calculated using David’s score [30] based on dyadic aggressive and submissive interactions, and the individuals were divided into one of the following three categories: high ranking (1–4), middle ranking (5–7), and low ranking (8–10) (Table 1).

### 2.2. Data Collection

The chimpanzees of the M group were followed by author SS and field assistants from 3 February to 18 April, from 23 June to 1 September 2018, and from 11 March to 24 May 2019. We employed the focal sampling method [31], in which a focal animal was followed for as long as possible every day from 7:10 am. We followed the first male we found in the morning unless we had followed that individual on the previous day. When we lost sight of the focal individual, we continued focal observation if the individual was found again within 30 min. However, if we could not find the individual after 30 min, we stopped the focal observation for that day. During the focal following, we recorded the composition of the party to which the focal animal belonged every 60 min using the 1 h party method [17,32,33]. We recorded the IDs of the individuals within visible range at the beginning of each hour and added new individuals that joined the party as they were observed until the end of the observation hour. Each hour-long observation was included in the analyses as a unit and described here as one observation hour unit (OHU) when the focal animal was successfully followed for more than 30 min within the hour of observation. SS recorded all observable intragroup aggression, social interactions, and events, including hunting and copulation, in each OHU using ad libitum sampling (see Hashimoto et al. 2001 for more details). In order to exclude the possibility of overestimating the data on behaviors that were easy to observe, we excluded all observation data for days with less than three OHUs.

#### 2.2.1. Definition of the Presence of Females with Maximum Swelling

Previous studies showed that male interest in females increases toward the end of the maximum swelling phase [34,35] and the probability of ovulation is the highest during that period [36]. The swelling status of the sexual skin of each female in each party was recorded as one of the following two categories: non-swelling and maximum swelling (herein after MS) [28]. Because the presence of females with MS could affect both male decisions to join or leave parties and male aggression rates, we defined all OHU on days when we observed females with MS at least once as “OHU under the presence of females with MS”.

#### 2.2.2. Fruit Abundance

We obtained data on fruit abundance from 12 parallel line transects, which were built 500 m apart and with a total length of 108 km. Twice a month, with intervals of approximately 15 days, a field assistant counted the number of clusters of newly fallen mature fruits that were found within 1 m on either side of each transect line. We calculated the fruit abundance index (FAI) for each census period by dividing the total number of clusters by the total length of transects (km) [37].

### 2.3. Statistical Analyses

In total, 677 OHUs were collected for all subjects with a mean of 67.7 ± 11.1 SD OHU (range: 53–90) for each individual (Table 1). The mean duration of OHU was 52.1 min (range: 30–60). We observed 177 dyadic aggressive interactions in 65 male–male dyads. As the number of aggression events in which the focal animal of the OHU was targeted was very few (18 interactions in total), we included all the aggressive interactions that occurred among subject males in the analyses. We used R (version 4.0.4; R Foundation for Statistical Computing, Vienna, Austria, http://www.r-project.org (accessed on 16 February 2021). We used the “glmmTMB” package [38] for all analyses using a generalized linear mixed model (GLMM). For each GLMM analysis, we selected the best model by using the “MuMIn” package [39].

#### 2.3.1. Male Attendance at Parties

To examine the factors affecting each male’s attendance at parties, we ran two series of GLMMs. For the first GLMM, we used one OHU as one data point. We entered 1 or 0, depending on whether the focal male was alone or with other males, respectively, in the OHU as the dependent variable using the “cbind” function and the error distribution “binomial.” The initial model included the dominance rank of the focal male (low vs. high and low vs. middle), the presence vs. absence of females with MS, FAI, the interaction between the dominance rank and presence of females with MS, and the interaction between the dominance rank and FAI as fixed factors. The ID of each focal male was used as a random effect to account for individual differences.

For the second GLMM, we entered the number of males in the party that the focal male attended in each OHU as the dependent variable using the error distribution “Poisson” function. The initial model included the dominance rank of the focal male, the presence vs. absence of females with MS, FAI, and the interaction between the dominance rank and MS as fixed factors. The ID of the focal male was used as a random effect. The results showed that the interaction between the dominance rank of the focal males and the presence vs. absence of females with MS had a significant effect. In order to interpret this result, we ran two more GLMMs separately for the dataset in the absence of females with MS and the presence of females with MS. We entered the number of males in the party that the focal male attended in each OHU as the dependent variable, using the error distribution “Poisson” function. The initial model included the dominance rank of the focal male and FAI as fixed factors. The ID of the focal male was used as a random effect.

#### 2.3.2. Frequency of Receiving Aggression

To examine the factors affecting the frequency of aggressive interactions in which subject males were targeted, we ran a GLMM with one OHU as one data point. We excluded data when focal animals ranged alone. We entered the number of aggressions the subject males received per hour as a dependent variable using the error distribution “zero-inflated Poisson” function. We included the length of OHU as an offset variable (to control for possible bias in the response variable). The initial model included the number of males in the party, presence vs. absence of females with MS, and dominance rank of the focal males (low-ranking vs. high-ranking and low-ranking vs. middle-ranking) as fixed factors. The ID of the male who received aggression was used as a random effect to account for individual differences.

## 3. Results

### 3.1. Male Attendance at Parties

The results of the first GLMM analysis (Table 2) showed that the dominance rank of males had a significant effect on the tendency of males to spend time alone. The probability of time spent alone by low-ranking males was significantly higher than that of males in higher rank classes. The presence of females with MS during the day of observation also had a significant and negative effect on the probability of the OHUs that males spent time alone, indicating that males attended parties more frequently on the days that females with MS were present irrespective of their dominance rank.

**Figure 1:**
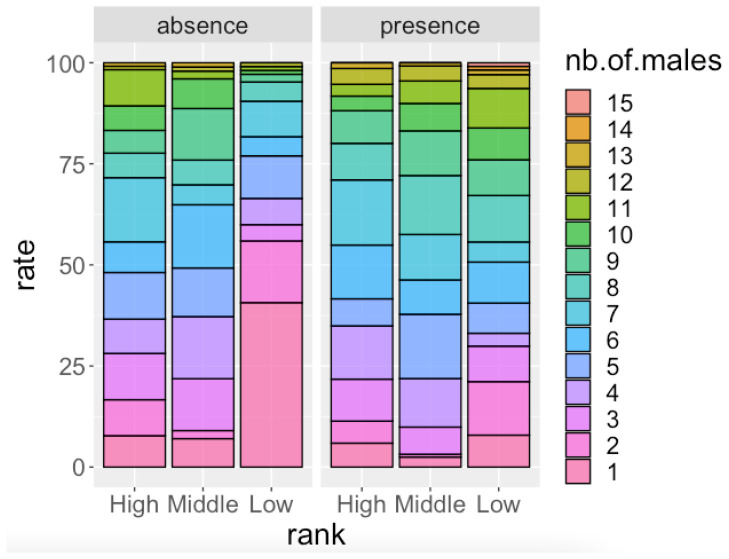
Participation ratio of males in relation to dominance rank on days when females with maximum sexual swelling (MS) were absent and on days when females with MS were present.

As mentioned in Section 2.3.1, in order to interpret the results of the second GLMM (Table 3) we ran two GLMM analyses separately for the dataset in the absence of females with MS and the presence of females with MS. The results of the GLMM analysis for the absence of females with MS (Table 4) showed that the dominance rank of focal males had a significant effect on the number of males in the party that the focal male attended; that is, high-ranking and middle-ranking males spent more time in larger parties than low-ranking males in the absence of females with MS. In the presence of females with MS, the effect of the dominance rank of males was not significant (Table 5). In both analyses (Table 4 and Table 5), the FAI had a significant and negative effect on the number of males in the party that the focal male attended. When the FAI was high, males tended to spend more time in smaller parties irrespective of their dominance rank. Figure 1 shows the proportion of party size that males of each dominance rank attended. During the days in which females with MS were absent, low-ranking males frequently ranged alone. During the days in which females with MS were present, males spent less time ranging alone and attended larger parties more frequently.

### 3.2. Frequency of Receiving Aggression

**Figure 2:**
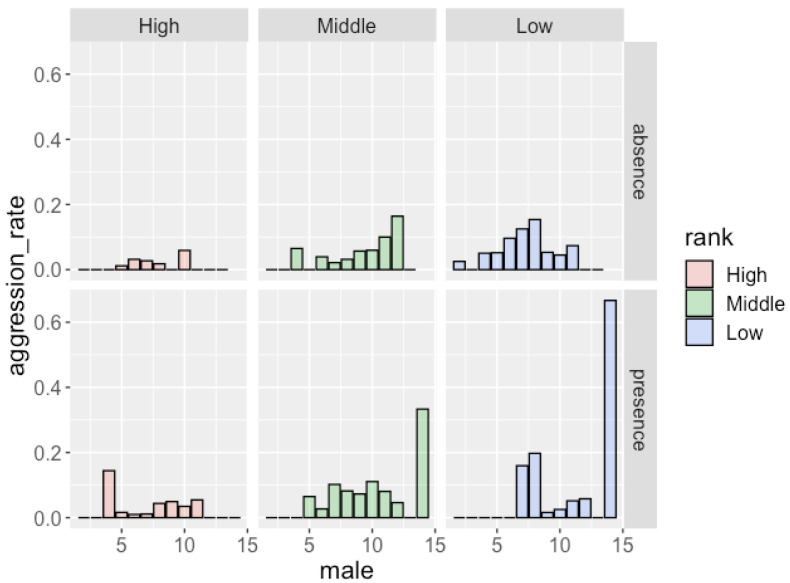
Frequency of aggression males received and the number of males in the party in the absence/presence of females with MS.

The results of the GLMM analysis (Table 6) showed that males received more aggression when attending larger parties. The dominance rank of males also had a significant effect on the frequency of aggression: low-ranking males received aggression more frequently than high-ranking males. The presence of females with MS did not have a significant effect on the occurrence of aggression. Figure 2 shows the frequency of aggression that males of each rank received. Although the effect of the dominance rank (middle-ranking males vs. low-ranking males) was not significant, low-ranking males received aggression more frequently than middle-ranking males (in total, 0.106 per hour for low-ranking males and 0.061 per hour for middle-ranking males).

## 4. Discussion

In this study, we investigated the influences of the dominance rank of each focal male, the presence of females with MS, and FAI on the size of parties that the focal males attended and the frequency of aggression that they received. In order to examine the three predictions we made from the hypothesis, we observed 10 adult male chimpanzees of the M group in Kalinzu using the focal animal sampling method.

We found that the dominance rank of males affected male tendencies of attendance at parties, with low-ranking males ranging alone and in parties with a less number of males more frequently than males of higher-rank classes. In the presence of females with MS, males of all ranks decreased their frequency of spending time alone and increased attendance at larger parties. In contrast, low-ranking males increased their frequency of ranging alone when females with MS were absent. These results suggest that low-ranking males tend to avoid attendance at parties, especially larger ones, unless they need to attend for access to receptive females.

The food abundance, represented by the FAI in this study, has been considered one of the main factors influencing the FFD of chimpanzee grouping [13,18,19,40]. However, other studies have shown that party size is not affected by the food abundance but by other factors such as the presence of receptive females [17,20,21,41]. Actually, in this study, the FAI, which represented the fruit food abundance, did not exert a significant influence on the tendency of males to range alone. Furthermore, males tended to attend smaller, rather than larger, parties when the FAI was higher. One explanation for this contradiction might be that the male chimpanzees of the M group tended to forage separately to avoid social tension when food was abundant in the forest and many food patches were available. In fact, in March 2018, when the FAI was the highest of all observation periods, chimpanzees often fed on the fruit of *Musanga leo-errerae*. As *Musanga* trees tended to be distributed sparsely and each tree could host one or a few individuals, we might have underestimated the number of individuals in the party. In contrast, in March 2019, when the FAI was the lowest, chimpanzees frequently fed on figs of *Ficus natalensis*. In Kalinzu, *F. natalensis* usually has a large canopy of 10–20 m diameter, and therefore, many individuals foraged on the same tree while forming a big party (Shibata, unpublished data). Similar results were reported in a previous study on the relationships among fruit abundance, fig abundance, and orangutan party size [42]. Thus, to investigate the relationship between food availability and party size of chimpanzees more precisely, we need to analyze the effect of the distribution of food patches as well as the FAI.

Overall, our results regarding the tendency of party attendance matched the predictions: (1) low-ranking males range alone or in small parties more frequently than higher-ranking males and (2) when there are no receptive females in the group, low-ranking males spend more time ranging alone than when there are receptive females in the group. The current results also matched prediction (3): low-ranking males receive aggression more frequently when attending large parties. The frequency of aggression increased with the number of males in the party and low-ranking males were attacked more frequently than higher-ranking males. However, the presence or absence of females with MS, which has been considered to affect the frequency of aggression among males [43,44], did not have a significant effect on the overall frequency of aggression toward focal males. This might be because the characteristic of male aggression changed in the absence of females with MS and the presence of those females. When the number of males was less than seven in the presence of females with MS, low-ranking males did not receive aggression and higher-ranking males received more aggression. This was most likely because the female with MS was monopolized by high-ranking males, and the low-ranking males did not attempt to approach her even when he was in the same party. In fact, low-ranking males stayed most of the time at the periphery of the party, away from the females with MS (Shibata, unpublished data). The FAI did not have any significant influence on the frequency of aggression in the parties.

The current study supports our hypothesis that the FFD may offer alternative tactics for low-ranking males to mitigate costs caused by disadvantages when competing for survival and reproduction against other adult males. The attendance of low-ranking males at parties seemed to be affected by the aggression they received during social gatherings, even in the absence of receptive females.

Male chimpanzees benefit from parties through opportunities for social and sexual interactions with other individuals. Affiliative interactions with other males during social gatherings are necessary for forging and maintaining social bonds, which are important for achieving a higher dominance status and for intergroup conflicts [45,46]. Furthermore, it is necessary for low-ranking males to attend large parties to seek mating opportunities with receptive females, who tend to be found in large parties as many males gather around them. However, low-ranking males receive limited benefits and are more likely to attract aggression from other males as they compete for food or receptive females or because higher-ranking males often show dominance displays toward low-ranking males in large parties. As observed in this study, the tendency of low-ranking males to receive aggression did not decrease even when there were no receptive females in the group. Therefore, it seemed to be more beneficial for low-ranking males to range alone, especially during the absence of receptive females.

A previous study on spider monkeys (*Ateles* spp.), which exhibit male philopatric social structures and strong fission–fusion tendencies similar to those of chimpanzees [47,48], suggested that the FFD of spider monkeys function as a form of conflict avoidance by mitigating the effect of food competition, both scramble and contest, and maintaining a low rate of aggression among adult females throughout the year [49]. Another study on spider monkeys reported that a young male individual was frequently observed alone after receiving aggression for several weeks before being killed [50]. Our findings suggest that male aggression of chimpanzees affects their dispersion tendencies, which are similar to those observed in spider monkeys.

However, in the present study, we did not investigate whether aggressive interactions trigger the leaves of chimpanzees, although aggressive interactions were most frequent toward low-ranking males. To understand the extent to which male aggression triggers dispersion, further studies are needed to assess aggressive interactions within parties and whether dispersion occurs after such interactions. In addition, we did not assess the possible effect of the existence of bonding partners or maternal brothers on the party attendance of individuals. These social factors are also likely to strongly affect individuals’ decisions on party attendance. Testing from a physiological perspective is also necessary to understand whether ranging alone is an adaptive choice for low-ranking males in terms of their stress levels. Hormonal analyses of individuals’ urinal/fecal cortisol levels would allow the estimation of stress levels in males when they range alone or in large/small parties. Research on the behavioral ecology of low-ranking chimpanzees, focusing on social and physiological aspects, is expected to further our understanding of the flexibility and complexity of the social structure of primates and other animals living in the FFD.

## 5. Conclusions

The present study results suggest that low-ranking males frequently ranged alone to avoid aggression from other males unless they attended parties to seek mating opportunities. The FFD seemed to offer alternative tactics for low-ranking males to mitigate costs caused by disadvantages when competing for survival and reproduction against other adult males. Further studies on the behavioral ecology of low-ranking chimpanzees, focusing on social and physiological aspects, are needed to better understand the flexibility and complexity of the social structure of primates and other animals living in the FFD.

## Figures and Tables

**Table 1:** Study subjects, dominance rank, birth year, age category, and the number of observation hour units (OHU).

Name (Abbreviation)	Dominance Rank	Birth Year	Age Class	OHU
Goku (GK)	Alpha	1993 *	Prime	90
Ponta (PO)	High	1995 *	Prime	53
Ichiro (IC)	High	1980s *	Old	76
Buru (BR)	High	1970s *	Old	65
Prince (PR)	Middle	1997 *	Prime	59
Taiki (TK)	Middle	1999	Young	77
Deo (DO)	Middle	1970s *	Old	62
Pietan (PT)	Low	2001	Young	65
Black (BL)	Low	1998 *	Young	72
Jo (JO)	Low	2000 *	Young	58

* age estimated.

**Table 2:** Results of GLMM analysis with males’ tendency of ranging alone compared to attending parties as the dependent variable.

	Variable Statistics					
Predictor Variables	Estimate	SE	z Value	*p* Value	Random Effects	Variance
Intercept	−0.61791	0.27047	−2.285	0.022 *	Subjects	0.096
Rank: Low vs. High	−1.63861	0.46026	−3.560	<0.001 ***		
Rank: Low vs. Middle	−2.13921	0.60170	−3.555	<0.001 ***		
MS: absence vs. presence	−1.84479	0.44766	−4.121	<0.001 ***		
FAI	0.14836	0.08311	1.785	0.074.		

* and *** indicate the significance at *p* = 0.05 and 0.001 levels, respectively.

**Table 3:** Results of GLMM analysis with the number of males in the party that the focal male attended as the dependent variable.

	Variable Statistics					
Predictor Variables	Estimate	SE	z Value	*p* Value	Random Effects	Variance
Intercept	1.8417	0.0587	31.394	<0.001 ***	Subjects	0.006
Rank: Low vs. High	0.1348	0.0660	2.043	0.041 *		
Rank: Low vs. Middle	0.1444	0.0693	2.082	0.037 *		
FAI	−0.0488	0.0080	−6.091	<0.001 ***		
MS: absence vs. presence	0.2723	0.0673	4.048	<0.001 ***		
Low vs. High: MS	−0.1928	0.0845	−2.282	0.022 *		

* and *** indicate the significance at *p* = 0.05 and 0.001 levels, respectively.

**Table 4:** Results of GLMM analysis with the number of males in the party that the focal male attended in the absence of females with MS as the dependent variable.

	Variable Statistics					
Predictor Variables	Estimate	SE	z Value	*p* Value	Random Effects	Variance
Intercept	1.7930	0.0688	26.068	<0.001 ***	Subjects	0.002
Rank: Low vs. High	0.1367	0.0660	2.071	0.038 *		
Rank: Low vs. Middle	0.1493	0.0694	2.150	0.031 *		
FAI	−0.0349	0.0129	−2.717	0.007 **		

*, **, and *** indicate the significance at *p* = 0.05, 0.01, and 0.001 levels, respectively.

**Table 5:** Results of GLMM analysis with the number of males in the party that the focal male attended in the presence of females with MS as the dependent variable.

	Variable Statistics					
Predictor Variables	Estimate	SE	z Value	*p* Value	Random Effects	Variance
Intercept	2.1468	0.0636	33.730	<0.001 ***	Subjects	0.004
Rank: Low vs. High	−0.0671	0.0675	−0.993	0.321		
Rank: Low vs. Middle	0.0267	0.0719	0.371	0.711		
FAI	−0.0578	0.0104	−5.553	<0.001 ***		

*** indicates the significance at *p* = 0.001 level.

**Table 6:** Results of GLMM analysis with the number of aggression that males received as the dependent variable.

	Variable Statistics					
Predictor Variables	Estimate	SE	z Value	*p* Value	Random Effects	Variance
Intercept	−4.5668	1.0009	−4.563	<0.001 ***	Subjects	0.005
Number of males	0.20789	0.0860	2.420	0.016 *		
Rank: Low vs. High	−1.60612	0.7848	−2.047	0.041 *		
Rank: Low vs. Middle	−0.52884	0.5923	−0.893	0.372		
MS: absence vs. presence	0.68867	0.5837	1.180	0.238		

* and *** indicate the significance at *p* = 0.05 and 0.001 levels, respectively.

## Data Availability

The data that support the findings of this study are available from the corresponding author on reasonable request.
